# (*E*)-*N*′-(3,3-Dimethyl-2,6-diphenyl­piperidin-4-yl­idene)isonicotinohydrazide

**DOI:** 10.1107/S1600536810040936

**Published:** 2010-10-20

**Authors:** C. Sankar, K. Pandiarajan, A. Thiruvalluvar, P. Gayathri

**Affiliations:** a Department of Chemistry, Annamalai University, Annamalai Nagar 608 002, Tamilnadu, India; b PG Research Department of Physics, Rajah Serfoji Government College (Autonomous), Thanjavur 613 005, Tamilnadu, India

## Abstract

In the title mol­ecule, C_25_H_26_N_4_O, the piperidine ring adopts a chair conformation, with the plane through the four coplanar atoms making dihedral angles of 84.76 (6), 82.28 (5) and 81.91 (6)° with the pyridine­ring and the phenyl rings at the 2 and 6 positions, respectively. The pyridine ring makes dihedral angles of 64.13 (8) and 10.75 (8)° with the phenyl rings at the 2 and 6 positions, respectively. The dihedral angle between the two phenyl rings is 53.57 (8)°. The phenyl rings and one of the methyl groups at position 3 have an equatorial orientation. In the crystal, mol­ecules are linked by N—H⋯O and C—H⋯O hydrogen bonds.

## Related literature

For the high bacteriostatic activity of isonicotinic acid hydrazide (INH) against mycobacterium tuberculosis, see: Hearn & Cynamon (2003 ▶). For Schiff bases of INH as anti­mycobacterial agents, see: Hearn *et al.* (2009 ▶). For a novel class of anti­mycobacterial agents, see: Jha & Dimmock (2006 ▶). For piperidin-4-ones as anti­bacterial agents, see: Srinivasan *et al.* (2006 ▶). For ring conformations, see: Cremer & Pople (1975 ▶).
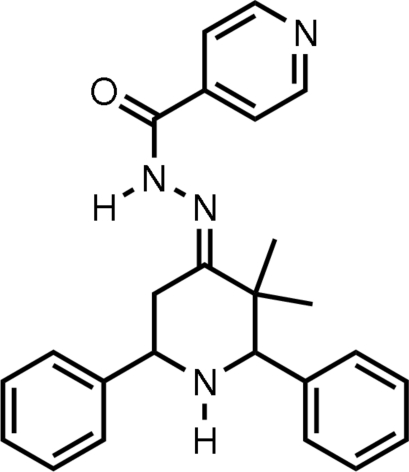


## Experimental

### 

#### Crystal data

C_25_H_26_N_4_O
*M* *_r_* = 398.50
Triclinic, 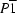

*a* = 6.2128 (1) Å
*b* = 12.8346 (3) Å
*c* = 15.0022 (3) Å
α = 65.293 (1)°
β = 78.823 (1)°
γ = 86.948 (1)°
*V* = 1065.62 (4) Å^3^
*Z* = 2
Mo *K*α radiation
μ = 0.08 mm^−1^
*T* = 293 K
0.22 × 0.18 × 0.16 mm

#### Data collection

Bruker Kappa APEXII CCD diffractometer
Absorption correction: multi-scan (*SADABS*; Bruker, 2004 ▶) *T*_min_ = 0.856, *T*_max_ = 1.000
25613 measured reflections
5420 independent reflections
3821 reflections with *I* > 2σ(*I*)
*R*_int_ = 0.023

#### Refinement

*R*[*F* ^2^ > 2σ(*F* ^2^)] = 0.044
*wR*(*F* ^2^) = 0.128
*S* = 1.01
5420 reflections
281 parameters
H atoms treated by a mixture of independent and constrained refinement
Δρ_max_ = 0.19 e Å^−3^
Δρ_min_ = −0.17 e Å^−3^

### 

Data collection: *APEX2* (Bruker, 2004 ▶); cell refinement: *SAINT-NT* (Bruker, 2004 ▶); data reduction: *SAINT-NT*; program(s) used to solve structure: *SHELXS97* (Sheldrick, 2008 ▶); program(s) used to refine structure: *SHELXL97* (Sheldrick, 2008 ▶); molecular graphics: *ORTEP-3* (Farrugia, 1997 ▶); software used to prepare material for publication: *PLATON* (Spek, 2009 ▶).

## Supplementary Material

Crystal structure: contains datablocks global, I. DOI: 10.1107/S1600536810040936/hg2726sup1.cif

Structure factors: contains datablocks I. DOI: 10.1107/S1600536810040936/hg2726Isup2.hkl

Additional supplementary materials:  crystallographic information; 3D view; checkCIF report

## Figures and Tables

**Table 1:** Hydrogen-bond geometry (Å, °)

*D*—H⋯*A*	*D*—H	H⋯*A*	*D*⋯*A*	*D*—H⋯*A*
N5—H5⋯O14^i^	0.89 (2)	2.10 (2)	2.9648 (17)	167.1 (17)
C5—H5*A*⋯O14^i^	0.97	2.56	3.4376 (17)	151

Symmetry code: (i) 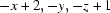.

**Fig. 1.**
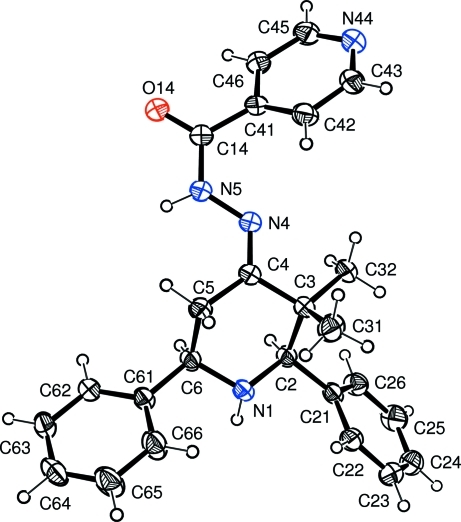
The molecular structure of the title compound, showing the atom-numbering scheme and displacement ellipsoids drawn at the 30% probability level. H atoms are shown as small spheres of arbitrary radius.

**Fig. 2.**
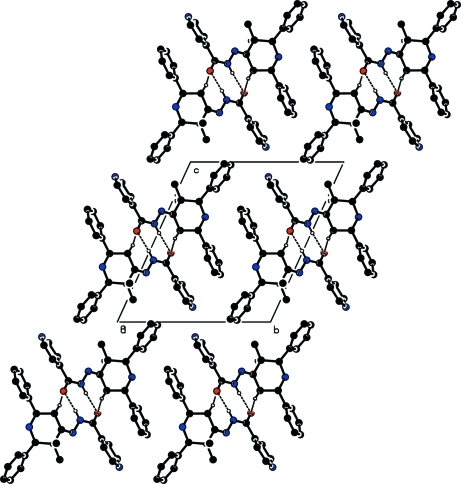
The packing of the title compound, viewed down the a axis. Dashed lines indicate hydrogen bonds. H atoms not involved in hydrogen bonding have been omitted.

## supplementary crystallographic information

### Comment

Isonicotinoyl hydrazide is the chief derivative of isonicotinic acid, possessing high bacteriostatic activity against mycobacteria of tuberculosis (Hearn & Cynamon (2003)) and is used for the treatment and localization of all forms of tuberculosis. Piperidin-4-one and its derivatives have been long known for wide spectrum of biological activities (Srinivasan *et al.*, (2006)). Schiff bases of Isonicotinic acid hydrazide (INH) (Hearn *et al.*, (2009)) have been shown high levels of activity against Mycobacterium tuberculosis *in vitro* and in tuberculosis-infected macrophages. Jha & Dimmock (2006) have reported a novel class of antimycobacterial agents. Due to the above importance, the crystal structure of the title compound (I) has been determined by X-ray diffraction.

In the title molecule, C_25_H_26_N_4_O, Fig.1., the piperidine ring adopts a chair conformation, with the plane through the four coplanar atoms (C2,C3,C5,C6) making a dihedral angle of 84.76 (6)°, 82.28 (5)° & 81.91 (6)° with the pyridine, phenyl rings at 2 & 6 respectively. The pyridine ring makes dihedral angles of 64.13 (8)° & 10.75 (8)° with the phenyl rings at 2 & 6 respectively. The dihedral angle between the two phenyl rings is 53.57 (8)°. The phenyl rings at position 2 & 6 and one of the methyl groups at position 3 have an equatorial orientation. The hydrazone double bond has E configuration about the >C═NNH bond. The puckering parameters (Cremer & Pople, 1975) of piperidine ring are q2=0.0644 (14) Å, q3=0.5856 (14) Å, Q=0.5890 (14) Å, θ=6.22 (14)° and φ=29.3 (13)°. Molecules are linked by N5—H5···O14 and C5—H5A···O14 hydrogen bonds (Table 1, Fig. 2).

### Experimental

A mixture of 3,3-dimethyl-r(2),c(6)-diarylpiperidin-4-one (0.28 g, 1 mmol), isoniazid (0.21 g, 1.5 mmol) and 0.5 ml of acetic acid in methanol medium was refluxed for three hours and then cooled to room temperature. The precipitate was filtered and washed with water. The crude product was recrystallized from ethanol. Colourless crystals were thus obtained in (0.298 g, 75%) yield. A single-crystal suitable for X-ray structure analysis was obtained by slow evaporation of a solution in a mixture of ethyl acetate and ethanol (1:1 *v*/*v*) at room temperature.

### Refinement

H1 at N1 and H5 at N5 atoms were located in a difference Fourier map and refined isotropically: N1—H1 = 0.90 (2) Å, N5—H5 = 0.89 (2) Å. Remaining H atoms were positioned geometrically and allowed to ride on their parent atoms, with C*sp*^2^—H = 0.93, *C*(methyl)—H = 0.96, *C*(methylene)—H = 0.97 and C(methine)—H = 0.98 Å; *U*_iso_(H) = k*U*_eq_(C), where k = 1.5 for methyl and 1.2 for all other H atoms.

### Crystal data

**Table:**

C_25_H_26_N_4_O	*Z* = 2
*M**_r_* = 398.50	*F*(000) = 424
Triclinic, *P*1	*D*_x_ = 1.242 Mg m^−^^3^
Hall symbol: -P 1	Melting point: 490 K
*a* = 6.2128 (1) Å	Mo *K*α radiation, λ = 0.71073 Å
*b* = 12.8346 (3) Å	Cell parameters from 9908 reflections
*c* = 15.0022 (3) Å	θ = 1.8–25.0°
α = 65.293 (1)°	µ = 0.08 mm^−^^1^
β = 78.823 (1)°	*T* = 293 K
γ = 86.948 (1)°	Prism, colourless
*V* = 1065.62 (4) Å^3^	0.22 × 0.18 × 0.16 mm

### Data collection

**Table:**

Bruker Kappa APEXII CCD diffractometer	5420 independent reflections
Radiation source: fine-focus sealed tube	3821 reflections with *I* > 2σ(*I*)
graphite	*R*_int_ = 0.023
ω and φ scan	θ_max_ = 28.6°, θ_min_ = 1.8°
Absorption correction: multi-scan (*SADABS*; Bruker, 2004)	*h* = −8→8
*T*_min_ = 0.856, *T*_max_ = 1.000	*k* = −17→17
25613 measured reflections	*l* = −20→20

### Refinement

**Table:**

Refinement on *F*^2^	Primary atom site location: structure-invariant direct methods
Least-squares matrix: full	Secondary atom site location: difference Fourier map
*R*[*F*^2^ > 2σ(*F*^2^)] = 0.044	Hydrogen site location: inferred from neighbouring sites
*wR*(*F*^2^) = 0.128	H atoms treated by a mixture of independent and constrained refinement
*S* = 1.01	*w* = 1/[σ^2^(*F*_o_^2^) + (0.0566*P*)^2^ + 0.2037*P*] where *P* = (*F*_o_^2^ + 2*F*_c_^2^)/3
5420 reflections	(Δ/σ)_max_ = 0.001
281 parameters	Δρ_max_ = 0.19 e Å^−^^3^
0 restraints	Δρ_min_ = −0.17 e Å^−^^3^

### Special details

**Table:**

Geometry. Bond distances, angles *etc*. have been calculated using the rounded fractional coordinates. All su's are estimated from the variances of the (full) variance-covariance matrix. The cell e.s.d.'s are taken into account in the estimation of distances, angles and torsion angles
Refinement. Refinement of *F*^2^ against ALL reflections. The weighted *R*-factor *wR* and goodness of fit *S* are based on *F*^2^, conventional *R*-factors *R* are based on *F*, with *F* set to zero for negative *F*^2^. The threshold expression of *F*^2^ > 2σ(*F*^2^) is used only for calculating *R*-factors(gt) *etc*. and is not relevant to the choice of reflections for refinement. *R*-factors based on *F*^2^ are statistically about twice as large as those based on *F*, and *R*- factors based on ALL data will be even larger.

### Fractional atomic coordinates and isotropic or equivalent isotropic displacement parameters (Å^2^)

**Table:**

	*x*	*y*	*z*	*U*_iso_*/*U*_eq_	
O14	1.03274 (18)	−0.14364 (9)	0.56827 (8)	0.0695 (4)	
N1	0.33045 (18)	0.26040 (10)	0.65579 (8)	0.0448 (3)	
N4	0.60062 (18)	−0.04110 (9)	0.69192 (8)	0.0467 (3)	
N5	0.74773 (19)	−0.05142 (10)	0.61377 (9)	0.0514 (4)	
N44	0.7572 (2)	−0.44715 (11)	0.90685 (10)	0.0642 (5)	
C2	0.36826 (19)	0.17468 (10)	0.75246 (9)	0.0414 (3)	
C3	0.3208 (2)	0.05259 (11)	0.76155 (9)	0.0448 (4)	
C4	0.4717 (2)	0.04288 (11)	0.67242 (9)	0.0443 (4)	
C5	0.4554 (2)	0.13676 (11)	0.57172 (9)	0.0496 (4)	
C6	0.4863 (2)	0.25405 (11)	0.57214 (9)	0.0444 (4)	
C14	0.8758 (2)	−0.14316 (11)	0.63200 (10)	0.0488 (4)	
C21	0.2393 (2)	0.20405 (10)	0.83490 (9)	0.0421 (4)	
C22	0.0312 (2)	0.24877 (12)	0.83080 (10)	0.0496 (4)	
C23	−0.0766 (3)	0.28184 (13)	0.90385 (11)	0.0580 (5)	
C24	0.0198 (3)	0.27101 (14)	0.98165 (11)	0.0636 (5)	
C25	0.2234 (3)	0.22530 (15)	0.98804 (12)	0.0665 (6)	
C26	0.3326 (2)	0.19245 (12)	0.91516 (10)	0.0542 (5)	
C31	0.0813 (2)	0.03536 (14)	0.75770 (13)	0.0622 (5)	
C32	0.3741 (3)	−0.03629 (12)	0.86027 (10)	0.0625 (5)	
C41	0.8240 (2)	−0.24660 (11)	0.72951 (10)	0.0439 (4)	
C42	0.6160 (2)	−0.28642 (12)	0.78285 (12)	0.0558 (5)	
C43	0.5917 (3)	−0.38594 (13)	0.86975 (12)	0.0631 (5)	
C45	0.9553 (3)	−0.40873 (13)	0.85467 (12)	0.0620 (5)	
C46	0.9973 (2)	−0.31085 (12)	0.76702 (11)	0.0538 (4)	
C61	0.4552 (2)	0.35141 (11)	0.47507 (9)	0.0443 (4)	
C62	0.6328 (2)	0.39763 (12)	0.39928 (10)	0.0529 (4)	
C63	0.6084 (3)	0.48708 (13)	0.30964 (11)	0.0613 (5)	
C64	0.4057 (3)	0.53062 (13)	0.29626 (11)	0.0644 (5)	
C65	0.2281 (3)	0.48566 (16)	0.37049 (13)	0.0766 (6)	
C66	0.2510 (3)	0.39566 (15)	0.45995 (12)	0.0689 (5)	
H1	0.345 (2)	0.3300 (15)	0.6547 (11)	0.059 (4)*	
H2	0.52434	0.18003	0.75368	0.0496*	
H5	0.794 (3)	0.0072 (16)	0.5558 (14)	0.075 (5)*	
H5A	0.56718	0.12780	0.52067	0.0595*	
H5B	0.31293	0.13153	0.55624	0.0595*	
H6	0.63545	0.26047	0.58215	0.0533*	
H22	−0.03622	0.25654	0.77826	0.0596*	
H23	−0.21595	0.31171	0.90010	0.0695*	
H24	−0.05261	0.29461	1.03008	0.0763*	
H25	0.28808	0.21638	1.04164	0.0798*	
H26	0.47123	0.16198	0.91995	0.0651*	
H31A	0.06299	−0.03715	0.75527	0.0934*	
H31B	−0.01240	0.03648	0.81622	0.0934*	
H31C	0.04326	0.09596	0.69918	0.0934*	
H32A	0.52223	−0.02334	0.86404	0.0938*	
H32B	0.27505	−0.02933	0.91505	0.0938*	
H32C	0.35859	−0.11194	0.86366	0.0938*	
H42	0.49352	−0.24676	0.76058	0.0670*	
H43	0.45002	−0.41165	0.90449	0.0757*	
H45	1.07448	−0.45049	0.87865	0.0745*	
H46	1.14085	−0.28823	0.73343	0.0646*	
H62	0.77117	0.36858	0.40818	0.0635*	
H63	0.72983	0.51746	0.25863	0.0736*	
H64	0.38929	0.59106	0.23630	0.0773*	
H65	0.09044	0.51550	0.36118	0.0919*	
H66	0.12831	0.36480	0.51013	0.0826*	

### Atomic displacement parameters (Å^2^)

**Table:**

	*U*^11^	*U*^22^	*U*^33^	*U*^12^	*U*^13^	*U*^23^
O14	0.0718 (7)	0.0506 (6)	0.0626 (7)	0.0153 (5)	0.0123 (5)	−0.0131 (5)
N1	0.0559 (6)	0.0363 (6)	0.0362 (6)	0.0060 (4)	−0.0079 (4)	−0.0103 (4)
N4	0.0519 (6)	0.0376 (6)	0.0438 (6)	0.0072 (4)	−0.0037 (5)	−0.0133 (5)
N5	0.0587 (7)	0.0419 (6)	0.0429 (6)	0.0118 (5)	−0.0016 (5)	−0.0118 (5)
N44	0.0746 (9)	0.0483 (7)	0.0572 (8)	0.0074 (6)	−0.0041 (6)	−0.0141 (6)
C2	0.0412 (6)	0.0404 (6)	0.0363 (6)	0.0043 (5)	−0.0081 (5)	−0.0099 (5)
C3	0.0481 (7)	0.0372 (6)	0.0407 (7)	0.0047 (5)	−0.0029 (5)	−0.0107 (5)
C4	0.0490 (7)	0.0369 (6)	0.0419 (7)	0.0051 (5)	−0.0069 (5)	−0.0127 (5)
C5	0.0582 (8)	0.0459 (7)	0.0394 (7)	0.0141 (6)	−0.0088 (6)	−0.0142 (6)
C6	0.0439 (6)	0.0435 (7)	0.0366 (6)	0.0047 (5)	−0.0081 (5)	−0.0080 (5)
C14	0.0518 (7)	0.0411 (7)	0.0492 (7)	0.0073 (5)	−0.0050 (6)	−0.0174 (6)
C21	0.0491 (7)	0.0350 (6)	0.0359 (6)	0.0016 (5)	−0.0086 (5)	−0.0085 (5)
C22	0.0531 (7)	0.0509 (8)	0.0420 (7)	0.0086 (6)	−0.0121 (5)	−0.0160 (6)
C23	0.0582 (8)	0.0569 (9)	0.0543 (8)	0.0116 (6)	−0.0061 (6)	−0.0219 (7)
C24	0.0834 (11)	0.0604 (9)	0.0473 (8)	0.0067 (8)	−0.0058 (7)	−0.0261 (7)
C25	0.0870 (11)	0.0714 (10)	0.0493 (9)	0.0103 (8)	−0.0256 (8)	−0.0286 (8)
C26	0.0597 (8)	0.0547 (8)	0.0498 (8)	0.0094 (6)	−0.0201 (6)	−0.0199 (7)
C31	0.0533 (8)	0.0591 (9)	0.0741 (10)	−0.0079 (7)	0.0000 (7)	−0.0316 (8)
C32	0.0834 (10)	0.0430 (8)	0.0414 (7)	0.0117 (7)	0.0001 (7)	−0.0047 (6)
C41	0.0488 (7)	0.0374 (6)	0.0468 (7)	0.0052 (5)	−0.0073 (5)	−0.0200 (5)
C42	0.0485 (7)	0.0436 (7)	0.0700 (10)	0.0042 (5)	−0.0108 (6)	−0.0190 (7)
C43	0.0585 (9)	0.0458 (8)	0.0712 (10)	−0.0030 (6)	0.0018 (7)	−0.0164 (7)
C45	0.0665 (9)	0.0547 (9)	0.0585 (9)	0.0187 (7)	−0.0169 (7)	−0.0172 (7)
C46	0.0484 (7)	0.0512 (8)	0.0569 (8)	0.0087 (6)	−0.0068 (6)	−0.0199 (7)
C61	0.0513 (7)	0.0392 (6)	0.0371 (6)	0.0028 (5)	−0.0091 (5)	−0.0107 (5)
C62	0.0575 (8)	0.0434 (7)	0.0467 (7)	0.0056 (6)	−0.0024 (6)	−0.0116 (6)
C63	0.0793 (10)	0.0447 (8)	0.0439 (8)	−0.0021 (7)	0.0041 (7)	−0.0093 (6)
C64	0.0909 (12)	0.0442 (8)	0.0468 (8)	0.0019 (7)	−0.0235 (8)	−0.0034 (6)
C65	0.0661 (10)	0.0710 (11)	0.0685 (11)	0.0096 (8)	−0.0279 (8)	0.0001 (9)
C66	0.0509 (8)	0.0719 (10)	0.0557 (9)	0.0046 (7)	−0.0109 (7)	0.0007 (8)

### Geometric parameters (Å, °)

**Table:**

O14—C14	1.2279 (17)	C61—C62	1.3717 (18)
N1—C2	1.4627 (17)	C62—C63	1.385 (2)
N1—C6	1.4578 (17)	C63—C64	1.367 (3)
N4—N5	1.3889 (17)	C64—C65	1.358 (2)
N4—C4	1.2744 (19)	C65—C66	1.384 (3)
N5—C14	1.345 (2)	C2—H2	0.9800
N44—C43	1.325 (2)	C5—H5A	0.9700
N44—C45	1.319 (2)	C5—H5B	0.9700
N1—H1	0.90 (2)	C6—H6	0.9800
N5—H5	0.89 (2)	C22—H22	0.9300
C2—C3	1.554 (2)	C23—H23	0.9300
C2—C21	1.5123 (18)	C24—H24	0.9300
C3—C4	1.5242 (18)	C25—H25	0.9300
C3—C32	1.5303 (19)	C26—H26	0.9300
C3—C31	1.5314 (18)	C31—H31A	0.9600
C4—C5	1.5063 (18)	C31—H31B	0.9600
C5—C6	1.530 (2)	C31—H31C	0.9600
C6—C61	1.5105 (18)	C32—H32A	0.9600
C14—C41	1.499 (2)	C32—H32B	0.9600
C21—C22	1.3882 (19)	C32—H32C	0.9600
C21—C26	1.3855 (18)	C42—H42	0.9300
C22—C23	1.380 (2)	C43—H43	0.9300
C23—C24	1.366 (2)	C45—H45	0.9300
C24—C25	1.369 (3)	C46—H46	0.9300
C25—C26	1.379 (2)	C62—H62	0.9300
C41—C42	1.3781 (19)	C63—H63	0.9300
C41—C46	1.3814 (19)	C64—H64	0.9300
C42—C43	1.381 (2)	C65—H65	0.9300
C45—C46	1.377 (2)	C66—H66	0.9300
C61—C66	1.382 (2)		
			
O14···N5^i^	2.9648 (17)	H2···H32A	2.4400
O14···H46	2.5900	H5···C5	2.65 (2)
O14···H5^i^	2.10 (2)	H5···H5A	2.0100
O14···H5A^i^	2.5600	H5···O14^i^	2.10 (2)
N4···C42	2.866 (2)	H5···C14^i^	3.037 (19)
N5···O14^i^	2.9648 (17)	H5A···N5	2.4800
N1···H31C	2.6200	H5A···H5	2.0100
N1···H22	2.6200	H5A···O14^i^	2.5600
N1···H66	2.5700	H5B···C31	2.8500
N4···H32A	2.6400	H5B···H31C	2.3400
N4···H32C	2.5300	H6···H2	2.3100
N4···H42	2.4700	H6···H62	2.3900
N5···H5A	2.4800	H22···N1	2.6200
N5···H42	2.8400	H22···C31	3.0200
N44···H43^ii^	2.7400	H23···H64^iv^	2.3400
C22···C31	3.330 (3)	H24···C45^vi^	2.8500
C26···C32	3.350 (2)	H26···H2	2.3600
C31···C22	3.330 (3)	H31A···H32C	2.5700
C32···C26	3.350 (2)	H31B···C21	2.8600
C42···N4	2.866 (2)	H31B···C22	2.8500
C46···C63^i^	3.596 (2)	H31B···H32B	2.4300
C46···C62^i^	3.546 (2)	H31C···N1	2.6200
C62···C46^i^	3.546 (2)	H31C···C5	2.8100
C63···C46^i^	3.596 (2)	H31C···H5B	2.3400
C5···H31C	2.8100	H32A···N4	2.6400
C5···H5	2.65 (2)	H32A···H2	2.4400
C14···H5^i^	3.037 (19)	H32B···C21	2.7400
C21···H32B	2.7400	H32B···C26	2.8900
C21···H31B	2.8600	H32B···H31B	2.4300
C22···H63^iii^	3.0600	H32C···N4	2.5300
C22···H31B	2.8500	H32C···H31A	2.5700
C22···H1	2.790 (14)	H32C···C24^v^	3.0800
C23···H64^iv^	3.0400	H42···N4	2.4700
C24···H32C^v^	3.0800	H42···N5	2.8400
C26···H64^iii^	3.1000	H42···H46^vii^	2.4300
C26···H32B	2.8900	H43···H45^vii^	2.5500
C31···H22	3.0200	H43···N44^ii^	2.7400
C31···H5B	2.8500	H45···H43^viii^	2.5500
C45···H63^i^	3.0100	H46···O14	2.5900
C45···H24^vi^	2.8500	H46···H42^viii^	2.4300
C46···H62^i^	3.0900	H46···C62^i^	2.9900
C62···H46^i^	2.9900	H62···H6	2.3900
C63···H1^iii^	2.66 (2)	H62···H65^viii^	2.5900
C64···H1^iii^	2.824 (18)	H62···C46^i^	3.0900
C66···H1	2.853 (15)	H63···C22^iii^	3.0600
H1···C22	2.790 (14)	H63···C45^i^	3.0100
H1···C66	2.853 (15)	H64···C23^iv^	3.0400
H1···C63^iii^	2.66 (2)	H64···C26^iii^	3.1000
H1···C64^iii^	2.824 (18)	H64···H23^iv^	2.3400
H2···H6	2.3100	H65···H62^vii^	2.5900
H2···H26	2.3600	H66···N1	2.5700
			
C2—N1—C6	112.55 (11)	C3—C2—H2	107.00
N5—N4—C4	118.90 (11)	C21—C2—H2	107.00
N4—N5—C14	119.82 (12)	C4—C5—H5A	110.00
C43—N44—C45	116.03 (15)	C4—C5—H5B	110.00
C2—N1—H1	108.0 (9)	C6—C5—H5A	110.00
C6—N1—H1	108.4 (9)	C6—C5—H5B	110.00
C14—N5—H5	114.1 (13)	H5A—C5—H5B	108.00
N4—N5—H5	123.4 (14)	N1—C6—H6	109.00
N1—C2—C21	109.36 (11)	C5—C6—H6	109.00
C3—C2—C21	115.32 (10)	C61—C6—H6	109.00
N1—C2—C3	109.55 (10)	C21—C22—H22	120.00
C2—C3—C4	105.43 (10)	C23—C22—H22	120.00
C31—C3—C32	109.43 (12)	C22—C23—H23	120.00
C2—C3—C32	108.92 (11)	C24—C23—H23	120.00
C4—C3—C31	109.77 (12)	C23—C24—H24	120.00
C2—C3—C31	112.07 (12)	C25—C24—H24	120.00
C4—C3—C32	111.19 (12)	C24—C25—H25	120.00
N4—C4—C3	116.53 (11)	C26—C25—H25	120.00
N4—C4—C5	127.81 (12)	C21—C26—H26	119.00
C3—C4—C5	115.62 (12)	C25—C26—H26	119.00
C4—C5—C6	109.92 (11)	C3—C31—H31A	109.00
N1—C6—C61	110.52 (11)	C3—C31—H31B	109.00
C5—C6—C61	111.96 (11)	C3—C31—H31C	109.00
N1—C6—C5	108.38 (11)	H31A—C31—H31B	109.00
O14—C14—C41	119.65 (13)	H31A—C31—H31C	109.00
N5—C14—C41	120.00 (12)	H31B—C31—H31C	109.00
O14—C14—N5	120.34 (13)	C3—C32—H32A	109.00
C2—C21—C22	122.00 (11)	C3—C32—H32B	109.00
C2—C21—C26	120.12 (12)	C3—C32—H32C	109.00
C22—C21—C26	117.80 (12)	H32A—C32—H32B	109.00
C21—C22—C23	120.70 (13)	H32A—C32—H32C	109.00
C22—C23—C24	120.49 (17)	H32B—C32—H32C	109.00
C23—C24—C25	119.76 (16)	C41—C42—H42	120.00
C24—C25—C26	120.11 (15)	C43—C42—H42	120.00
C21—C26—C25	121.13 (13)	N44—C43—H43	118.00
C42—C41—C46	116.93 (13)	C42—C43—H43	118.00
C14—C41—C42	125.26 (12)	N44—C45—H45	118.00
C14—C41—C46	117.73 (12)	C46—C45—H45	118.00
C41—C42—C43	119.17 (14)	C41—C46—H46	120.00
N44—C43—C42	124.22 (16)	C45—C46—H46	120.00
N44—C45—C46	124.32 (16)	C61—C62—H62	120.00
C41—C46—C45	119.32 (14)	C63—C62—H62	120.00
C6—C61—C62	119.72 (12)	C62—C63—H63	120.00
C6—C61—C66	121.71 (12)	C64—C63—H63	120.00
C62—C61—C66	118.57 (13)	C63—C64—H64	120.00
C61—C62—C63	120.77 (13)	C65—C64—H64	120.00
C62—C63—C64	119.93 (15)	C64—C65—H65	120.00
C63—C64—C65	120.03 (15)	C66—C65—H65	120.00
C64—C65—C66	120.35 (18)	C61—C66—H66	120.00
C61—C66—C65	120.35 (16)	C65—C66—H66	120.00
N1—C2—H2	107.00		
			
C6—N1—C2—C3	−66.16 (13)	N1—C6—C61—C62	147.45 (14)
C6—N1—C2—C21	166.56 (11)	N1—C6—C61—C66	−32.7 (2)
C2—N1—C6—C5	62.66 (13)	C5—C6—C61—C62	−91.63 (16)
C2—N1—C6—C61	−174.31 (11)	C5—C6—C61—C66	88.18 (17)
C4—N4—N5—C14	−175.94 (13)	O14—C14—C41—C42	−144.04 (16)
N5—N4—C4—C3	−177.47 (12)	O14—C14—C41—C46	32.6 (2)
N5—N4—C4—C5	0.2 (2)	N5—C14—C41—C42	34.5 (2)
N4—N5—C14—O14	−166.63 (13)	N5—C14—C41—C46	−148.87 (14)
N4—N5—C14—C41	14.9 (2)	C2—C21—C22—C23	−175.68 (14)
C45—N44—C43—C42	−0.9 (3)	C26—C21—C22—C23	0.9 (2)
C43—N44—C45—C46	0.6 (3)	C2—C21—C26—C25	176.00 (15)
N1—C2—C3—C4	57.28 (12)	C22—C21—C26—C25	−0.6 (2)
N1—C2—C3—C31	−62.09 (14)	C21—C22—C23—C24	−0.1 (3)
N1—C2—C3—C32	176.68 (11)	C22—C23—C24—C25	−1.1 (3)
C21—C2—C3—C4	−178.86 (10)	C23—C24—C25—C26	1.3 (3)
C21—C2—C3—C31	61.77 (14)	C24—C25—C26—C21	−0.4 (3)
C21—C2—C3—C32	−59.46 (14)	C14—C41—C42—C43	177.21 (15)
N1—C2—C21—C22	37.55 (17)	C46—C41—C42—C43	0.5 (2)
N1—C2—C21—C26	−138.95 (13)	C14—C41—C46—C45	−177.74 (15)
C3—C2—C21—C22	−86.41 (16)	C42—C41—C46—C45	−0.8 (2)
C3—C2—C21—C26	97.09 (15)	C41—C42—C43—N44	0.4 (3)
C2—C3—C4—N4	124.14 (13)	N44—C45—C46—C41	0.2 (3)
C2—C3—C4—C5	−53.79 (14)	C6—C61—C62—C63	−179.90 (14)
C31—C3—C4—N4	−114.98 (15)	C66—C61—C62—C63	0.3 (2)
C31—C3—C4—C5	67.09 (16)	C6—C61—C66—C65	179.38 (17)
C32—C3—C4—N4	6.26 (18)	C62—C61—C66—C65	−0.8 (3)
C32—C3—C4—C5	−171.68 (12)	C61—C62—C63—C64	0.4 (3)
N4—C4—C5—C6	−124.15 (15)	C62—C63—C64—C65	−0.5 (3)
C3—C4—C5—C6	53.51 (15)	C63—C64—C65—C66	0.0 (3)
C4—C5—C6—N1	−53.75 (13)	C64—C65—C66—C61	0.7 (3)
C4—C5—C6—C61	−175.90 (10)		

Symmetry codes: (i) −*x*+2, −*y*, −*z*+1; (ii) −*x*+1, −*y*−1, −*z*+2; (iii) −*x*+1, −*y*+1, −*z*+1; (iv) −*x*, −*y*+1, −*z*+1; (v) −*x*, −*y*, −*z*+2; (vi) −*x*+1, −*y*, −*z*+2; (vii) *x*−1, *y*, *z*; (viii) *x*+1, *y*, *z*.

### Hydrogen-bond geometry (Å, °)

**Table:**

*D*—H···*A*	*D*—H	H···*A*	*D*···*A*	*D*—H···*A*
N5—H5···O14^i^	0.89 (2)	2.10 (2)	2.9648 (17)	167.1 (17)
C5—H5A···O14^i^	0.97	2.56	3.4376 (17)	151

Symmetry codes: (i) −*x*+2, −*y*, −*z*+1.
